# Expression of TNF-α and RANTES in drug-induced human gingival overgrowth

**DOI:** 10.4103/0253-7613.66842

**Published:** 2010-06

**Authors:** Tamilselvan Subramani, Loganathan Dhanaraj, Kamatchiammal Senthilkumar, Soundararajan Periasamy, Georgi Abraham, Suresh Rao

**Affiliations:** Department of Periodontics, Sri Ramachandra Dental College and Hospital, Porur, Chennai - 600 116, India; 1 NEERI, Council of Scientific and Industrial Research, Taramani, Chennai - 600 113, India; 2 Department of Nephrology, Sri Ramachandra Medical College and Research Institute, Sri Ramachandra University, Porur, Chennai - 600 116, India

**Keywords:** Cyclosporine, overgrowth, periodontitis, RANTES, TNF-α

## Abstract

**Objectives:**

Regulated on activation, normal T cell expressed and secreted (RANTES) is a chemokine that is produced by fibroblasts, lymphoid and epithelial cells of the mucosa in response to various external stimuli. RANTES expression has been demonstrated in a variety of diseases characterized by inflammation, including asthma, transplantationassociated accelerated atherosclerosis, endometriosis and fibrosis. RANTES mRNA is quickly up-regulated by tumor necrosis factor (TNF)-α stimulation. Cyclosporine A (CsA) is widely used in organ transplant patients, often causing various side-effects including gingival overgrowth, which is fibrotic in nature. This study was carried out to assess the mRNA expression of TNF-α and RANTES in healthy individual, chronic periodontitis and CsAinduced gingival overgrowth tissues.

**Materials and Methods:**

Gingival tissue samples were collected from chronic periodontitis, CsAinduced gingival overgrowth patients and healthy individuals. Total RNA was isolated and reverse transcription polymerase chain reaction (RT-PCR) was performed for TNF-α and RANTES expression.

**Results:**

The results suggest that CsAinduced gingival overgrowth tissues expressed significantly increased TNF-α and RANTES compared to control and chronic periodontitis.

**Conclusion:**

The findings of the present study suggest that CsA can modify the expression of TNF-α and RANTES in drug-induced human gingival overgrowth.

Regulated on activation, normal T cell expressed and secreted (RANTES) is a member of the C-C chemokine subfamily and is produced by fibroblasts, lymphoid and epithelial cells of the mucosa in response to various external stimuli.[1–3] RANTES expression has been demonstrated as an important proinflammatory mediator in a variety of diseases characterized by inflammation, including asthma, transplantation-associated accelerated atherosclerosis, endometriosis and fibrosis.[4,5] RANTES mRNA is quickly up-regulated by tumor necrosis factor (TNF)-a stimulation.[6] Most organs of the body appear to be affected by TNF-α, and the cytokine serves a variety of functions, many of which are not yet fully understood. The cytokine possesses both growth-stimulating and inhibitory properties, and it appears to have self-regulatory properties as well.

Gingivitis is an inflammatory disease of bacterial origin initiated and perpetuated by pathogenic bacteria. The disease is characterized by infiltration of inflammatory cells like neutrophils, macrophages, etc.[7,8] Periodontitis results from the inflammatory response to bacterial challenge in the gingival crevicular area. Periodontal pathogens stimulate release of TNF-α from gingival macrophages.[9] TNF-α is an important proinflammatory cytokine present within the inflamed gingiva, and it regulates the responses of neutrophils to periodontopathogenic bacteria.[10] Increased tissue volume and cell numbers are characteristic features of hyperplasic pathologies that occur in many tissues, including kidney, liver, prostate and gingiva. This condition is becoming a far more widespread problem because of the greater number of people being treated with certain drugs that cause gingival overgrowth. These include the antiepileptic drug phenytoin, the calcium channel-blocking drug nifedipine and, most notably, cyclosporin A (CsA), which is very widely used as an immunosuppressant to prevent rejection following organ transplantation.[11–13] CsAinduced gingival overgrowth can be characterized by an increase in the synthesis or decrease in the degradation of extracellular matrix components of the gingival connective tissue and/or a combination of both these mechanisms. It has been assumed that CsA alters gingival fibroblast activity through effects on various cytokines.[14] Therefore, the objective of this study was to investigate the expression of chemokine RANTES and proinflammatory cytokine TNF-α in gingival tissue biopsies from chronic periodontitis and CsAinduced gingival overgrowth patients.

## Materials and Methods

### Materials

Gingival tissues were collected at the time of resective surgery from eight chronic periodontitis and eight CsAinduced gingival overgrowth patients aged 40-60 years. Eight control samples were also obtained from healthy individuals who had undergone orthodontic treatment. Drug-induced gingival overgrowth tissue samples were obtained from CsA-medicated organ transplant recipients during gingivectomies required to treat drug-induced gingival overgrowth. All the samples represented a moderate to severe degree of gingival overgrowth. All the diseased samples expressed clinical signs of inflammation and were taken from sites with a pocket depth >6 mm, with evidence of bleeding on probing. The control subjects were systemically and periodontally healthy without gingival inflammation and showed no evidence of bleeding on probing, with a pocket depth <4 mm. All the patients and healthy subjects were nonsmokers from enquiry on personal history. People who were medically compromised, who had antibiotic intake within 6 months and who had undergone any periodontal therapy were excluded from the study. Informed consent was obtained from all subjects and the study was approved by the Institutional Review Board of Sri Ramachandra Medical College and Research Institute.

### Analysis of TNF-α and RANTES mRNA

The total RNA was isolated from the gingival tissues by a single-step, acid guanidine thiocyanate-phenol-chloroform extraction method.[15] The total RNA was transcribed to cDNA using a First Strand cDNA synthesis Kit (Qbiogene, Chandigarh, India) for RT-PCR according to manufacturer instructions. The TNF-α and RANTES primers were designed from the known sequences, as TNF-α: 5’-TCTTCTCGAACCCCGAGTGA-3’ (sense) and 5’-CCTCTGATGGCACCACCAG-3’ (antisense); RANTES: 5’-ACAGGTA CCATGAAGGTCTC-3’ (sense) and 5’-TCCTAGCTCATCTCCAAAGA-3’ (antisense). The primers were predicted to amplify 229 and 269 base pairs, respectively. As a positive control, the β-actin primer, designed as 5’-AAGGATTCCT ATGTGGGC-3’ (sense) and 5’-CATCTCTTGCTCGAAGTC-3’ (antisense), was predicted to amplify a 300 base pair DNA fragment. The amplification profile was as follows: initial denaturing at 94°C for 5 min followed by denaturing at 94°C for 90 s, annealing at (56°C for TNF-α and β-actin, 61C for RANTES) for 60 s, extension at 72°C for 60 s. The cDNA was amplified for 30 cycles for TNF-α, 40 cycles for RANTES and 36 cycles for β-actin, followed by a step of 7 min at 72°C to extend the partially amplified products. The PCR products were electrophoresed on 1.5% agarose gel and visualized by ethidium bromide staining. The gels were photographed and their image data were analyzed using 1D analysis software. The relative amount of each TNF-α and RANTES gene expression was calculated as the ratio of the individual TNF-α and RANTES to the intensity of β-actin gene products as the control. The relative expression of each TNF-α and RANTES gene from control, diseased and drug-induced gingival overgrowth tissues were compared.

### Statistical analysis

Results are expressed as mean ± SD. Statistical analysis was performed by ANOVA. A value of *P* <0.05 was considered statistically significant.

## Results

**Figure 1:**
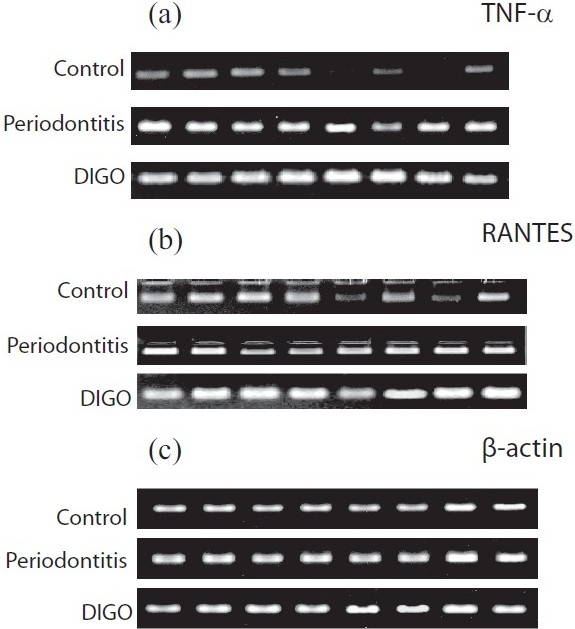
Expression of tumor necrosis factor (TNF)-α and regulated on activation, normal T cell expressed and secreted (RANTES) in periodontal tissues by RT-PCR analysis. The mRNA levels of TNF-α, RANTES and β-actin are shown. (a) TNF-α, (b) RANTES, (c) β-actin expressions were shown in cyclosporine-A-induced gingival overgrowth (DIGO), periodontitis and normal healthy individuals (control)

**Figure 2:**
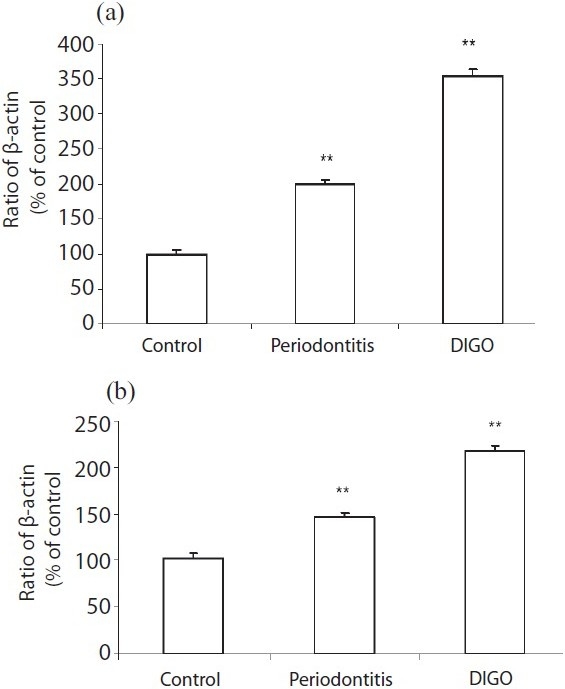
The mRNA expression of tumor necrosis factor (TNF)-α and regulated on activation, normal T cell expressed and secreted (RANTES) in human gingival samples obtained from patients with cyclosporin-A-induced gingival overgrowth (DIGO), periodontitis and normal healthy subjects (control). β-actin was used as the internal control for PCR. Each bar gives the mean and standard deviation of eight cDNA samples. (a) TNF-α mRNA expression and (b) RANTES mRNA expression in control, periodontitis and DIGO. Both TNF-α and RANTES were expressed in the control, but the level of expression in DIGO and periodontitis was significantly increased compared to the control

The expression of TNF-α, RANTES and β-actin in human gingival tissues with chronic periodontitis and drug-induced gingival overgrowth was examined. The amount of β-actin PCR product was used as the standard for the analysis of TNF-α and RANTES mRNA expressions. TNF-α and RANTES mRNA were not only expressed in the drug-induced gingival overgrowth and periodontitis but also, weakly, in the uninflamed gingival tissues [Table 1]. The expression of TNF-α and RANTES in periodontal tissues by RT-PCR analysis are shown in Figure 1a–c. The level of the TNF-α and RANTES mRNA expression from periodontitis and drug-induced gingival overgrowth tissue samples were increased compared with that in the uninflamed tissues [Figure 2a–b]. On the other hand, drug-induced gingival overgrowth tissue samples showed significantly increased expression than the chronic periodontitis tissue samples.

**Table 1:** The mRNA expression of TNF-α and RANTES in human gingival samples obtained from patients with cyclosporin-A-induced gingival overgrowth (DIGO), periodontitis and normal healthy subjects (control). TNF-α and RANTES were significantly expressed in periodontitis and DIGO compared to controls

*Cytokine/ chemokine*	*Condition*	*Number of tissue samples*	*Mean value of mRNA expression*	*Standard deviation*	*Standard error*	*Significance between groups*
TNF-α	Control	10	109.71	14.8955	4.7103	-
	Periodontitis	10	146.25	24.9785	8.8312	0.01
	DIGO	10	212.95	60.8872	19.2542	0.001
RANTES	Control	10	99.50	16.3859	5.1817	-
	Periodontitis	10	192.89	27.8103	8.7944	0.001
	DIGO	10	367.32	20.4028	7.2135	0.000

## Discussion

Studies of chemokines are currently being undertaken to further understand their role in the pathogenesis of a number of diseases because of their potential use as targets for therapy. The present experiment is performed to study the role of RANTES and the cytokine TNF-α expression in human gingival tissues with chronic periodontitis and CsAinduced gingival overgrowth compared with normal healthy gingival tissues. All control tissues expressed a small amount of RANTES and proinflammatory cytokine TNF-α. Studies have shown that the numbers of healthy gingival tissue sections identified positive for RANTES expression.[16,17] Several reports have suggested a relationship between the progression of chronic periodontitis and the expression of interleukin-1 (IL-1), IL-6, IL-8 and TNF-α in the gingival tissue.[18,19] We found a significant increase of RANTES expression and TNF-α expression in inflammation as compared to control tissues. RANTES attracts monocytes, eosinophils, basophils, NK cells, and T cells during inflammation and immune response, arguing for a role of this chemokine in chronic periodontitis.[20,21] Periodontal pathogens stimulate release of TNF-α from gingival macrophages.[22] Studies have shown that macrophage chemotactic protein-1 (MCP-1), macrophage inflammatory protein (MIP)-1α, MIP-1β and RANTES-producing cells were found to be present in inflamed human gingival tissues.[23] Compared to control and inflammation, TNF-α and RANTES expressions were significantly increased in CsAinduced gingival overgrowth. Normally, RANTES expression is increased following cellular activation of fibroblasts, T cells, monocytes and endothelial and epithelial cells.[24] Many cell types, including fibroblasts, epithelial cells and monocytes/macrophages, express RANTES in the early hours of stimulation by proinflammatory stimuli such as TNF-α.[25] Experimental murine studies indicate that development of fibrosis is influenced by several counter-regulatory cytokines, including IL-4, IL-13, TNF-α and interferon (IFN)-γ.[26] Studies have proven that cases of severe portal fibrosis were associated with low levels of IFN-γ and high levels of TNF-α.[27] In this study, we focused on associations between TNF-α and RANTES in terms of both their constitutive levels and their responses in drug-induced gingival overgrowth. TNF-α has been reported to inhibit the rates of collagen formation,[27] affecting rates of wound healing,[28] possibly by blockage of procollagen synthesis by the resident fibroblasts.[29] However, TNF-α is a potent inducer of the synthesis of matrix metalloproteinases (MMP) involved in the metabolism of collagen found in the extracellular matrix. Whereas this function of TNF-α is likely to be important in the pathogenesis of tissue-destructive diseases such as druginduced gingival overgrowth, it is clear that the production of TNF-α induced by RANTES relates to the risk of fibrosis. TNF-α is reported to promote fibrosis.[30] Inhibition of net collagen accumulation within tissues in the presence of TNF-α has also been attributed to its enhancement of collagenase activity.[31] These data suggest that TNF-α may have dual effects on collagenase protein synthesis. The proinflammatory cytokine TNF-α showed increased expression in druginduced gingival overgrowth, which could also contribute to the enhanced effect of cyclosporine on collagenous protein synthesis by gingival fibroblasts. Although TNF-α and cyclosporine are both present within overgrowth gingiva, there are no studies of their potential synergistic effects on gingival connective tissue metabolism. Various studies have demonstrated the hypothesis that TNF-α increases collagen accumulation and proliferation in intestinal myofibroblasts.[32,33] Our findings in gingival fibrosis correlated with other fibrotic conditions of elevated TNF-α, RANTES mRNA and protein production found in cystic fibrosis. Our results contrast with those of a study by Schwiebert showing no detectable RANTES expression from cultured cystic fibrosis (CF) surface epithelial cell lines.[34] One possible explanation for this difference is that signaling transduction pathways may be differently implicated in cystic fibrosis bronchial epithelial cells and bronchial epithelial cell lines. Our observations suggest that RANTES represents an important mediator of tissue mast cell recruitment in the setting of inflammatory reactions. These reports suggest a biological mechanism for the contribution of cyclosporine to gingival overgrowth, i.e. it reverses the inhibition of fibroblast proliferation and rate of collagen synthesis by gingival fibroblasts coincident to the expression of TNF-α and enhances these parameters in the presence of RANTES. However, the exact mechanism of differential regulation and polarized secretion of TNF-α and RANTES is a broad and complex subject that cannot be addressed extensively in the present work. The results from the present analysis offer new insights into the complexity of the disease process in humans.
